# A Scoping Review to Identify Clinical Signs, Symptoms and Biomarkers Reported in the Literature to Be Indicative of Biofilm in Chronic Wounds

**DOI:** 10.1111/iwj.70181

**Published:** 2025-05-19

**Authors:** John D. Ivory, Laure Perrier, Akke Vellinga, Duygu Sezgin, Chloe M. Hobbs, Cathal Ffrench, Patricia M. Coutts, James P. O'Gara, Georgina Gethin

**Affiliations:** ^1^ School of Nursing & Midwifery, Áras Moyola University of Galway Galway Ireland; ^2^ Alliance for Research & Innovation in Wounds (ARIW), School of Nursing & Midwifery, Áras Moyola University of Galway Galway Ireland; ^3^ Ontario Hospital Association (OHA) Toronto Ontario Canada; ^4^ School of Public Health, Physiotherapy & Sports Science, University College Dublin, Health Sciences Centre Dublin 4 Ireland; ^5^ Microbiology, School of Biological and Chemical Sciences University of Galway Galway Ireland; ^6^ Wounds Canada North York Ontario Canada; ^7^ Monash Nursing & Midwifery, Monash University, Clayton Campus Clayton Victoria Australia

**Keywords:** biofilms, chronic wounds, scoping review, signs and symptoms, wound healing

## Abstract

The objective of this review was to identify clinical signs/symptoms reported in the literature to be indicative of biofilm in chronic wounds. The Preferred Reporting Items for Systematic Reviews and Meta‐Analysis extension for Scoping Reviews, and the Joanna Briggs Institute Evidence Synthesis manual guided review conduct. Any article/study type reporting signs/symptoms of biofilm in adults with venous, diabetic, pressure and/or mixed arterial/venous ulcers was eligible. Medline, Embase, CINAHL, Cochrane CENTRAL and the Bielefeld Academic Search Engine were searched. Titles/abstracts and full‐text articles were screened against eligibility criteria. One‐hundred and eleven reports of 109 articles were included. They provided 830 accounts of clinical signs/ symptoms being indicative of biofilm. These were categorised into 26 statements. Visual indicators such as a shiny, slimy layer on a non‐healing wound surface quickly reforming in the absence of frequent cleansing or debridement represented 24% of accounts, followed by failed response to antimicrobial therapies (15%), and failure of wound to close or progress to healing despite optimal management strategies (13%). Wound duration > 6 weeks and extreme tolerance to host defences represented 1% of accounts. Clinical signs/symptoms are recommended and used as indicators of biofilm presence in chronic wounds but with little supporting validation data.

**Summary**

- Biofilm has been implicated in the delayed healing of chronic wounds and in the absence of convenient, bedside‐friendly diagnostic tests to confirm biofilm presence, clinicians often rely on clinical sign and symptom indicators.
- This scoping review, guided by the Preferred Reporting Items for Systematic Reviews and Meta‐Analyses extension for Scoping Reviews (PRISMA‐ScR) statement and the Joanna Briggs Institute Manual for Evidence Synthesis, aimed to identify and catalogue which clinical signs and symptoms are being reported in the literature to indicate the presence of biofilm in chronic wounds.
- One‐hundred and eleven reports of 109 full‐text articles eligible for this review provided 830 accounts of a sign or symptom said to be indicative of biofilm. We refined and categorised these into a list of 26 sign and symptom statements.
- Use of clinical signs and symptoms to confirm presence of biofilm in chronic wounds is commonly recommended, yet very little supporting validation work appears to exist.

## 1 Introduction

When the process of wound healing that incorporates phases of haemostasis, inflammation, proliferation and remodelling/maturation becomes disordered or interrupted, injuries to the skin may exhibit delayed or stalled healing and devolve into chronic wounds [1]. These interruptions often stem from comorbidities such as venous insufficiency, arterial disease, diabetes, neuropathies, and unresolved pressure [2, 3]. Genetic factors, radiation and immunological factors can also contribute to chronic wounds [4].

The global prevalence of chronic wounds (mixed aetiologies) has been calculated via meta‐analysis to be 2.21 per 1000 population while the pooled prevalence of chronic leg ulcers has been estimated to be 1.5 per 1000 population, and the burdens they cause to patients and healthcare systems in terms of finance and quality of life are well recognised and documented [5, 6, 7, 8].

Microorganisms are present in all wounds in what can initially be a host‐microbe relationship with no host reaction or delay in healing observed. Whether this initial presence develops along a continuum of colonisation and local wound infection through to spreading and systemic infection is contingent on factors such as increasing bacterial burden, the species of microbes present and inter‐species synergy, and the robustness of the host immune system [9].

If not diagnosed and treated promptly, infections in chronic wounds can lead to delayed healing, imposed limits on physical, social and psychological functioning, and a prolonged clinical condition may result [10, 11]. Complications secondary to infected pressure ulcers include endocarditis and meningitis [12], and for persons with diabetic foot ulceration, the risk of hospitalisation increases 50‐fold if a wound becomes infected and that of lower‐extremity amputation increases by a factor of 150 [13]. Treatment strategies for chronic wound infections include debridement to remove debris and necrotic tissue along with appropriate use of topical antiseptics and/or systemic antibiotics, but with rates of antibiotic resistance being directly related to level of antibiotic use, clinicians are recommended to apply principals of antimicrobial stewardship to patient care [14].

Biofilms are a composed of sessile bacterial cells existing as mono or multi‐species communities encased in a self‐produced exopolymeric substance that affix themselves to biotic or abiotic surfaces [15]. They are morphologically and physiologically distinct from the planktonic phenotype and are thought to be the prevailing microbial lifestyle in most natural habitats [16, 17]. Biofilms are estimated to be responsible for up to 80% of all human infections [18]. They are thought to be present in 6% of acute wounds but can be found in 60% to 78% of chronic wounds, possibly in over 90% [19, 20, 21]. They engage in multiple complex survival strategies that are both inherent to the biofilm phenotype and a result of biofilm‐host interactions [22], and after controlling for factors such as ensuring adequate compression, restoring arterial inflow, offloading and management of underlying systemic disease, they may be “the most important single cause of persistent, delayed healing” [23].

Biofilms can be difficult to diagnose and treat, and strategies for both are not without controversy and uncertainty [24]. Microbial culturing is the standard method for identifying pathogenic organisms colonising wounds in clinical settings, but can underestimate the degree of bacterial colonisation and number of species present in a wound in terms of the biofilm phenotype [25, 26]. This may be due to presence of viable cells in a biofilm that exist in a non‐culturable state, that is, they may be dormant or slow‐growing variants that do not form colonies under laboratory culture conditions, or biofilms located in deeper tissue [25, 26, 27]. Advanced microscopy is an alternative option but is not freely available [27]. Clinicians can rely on clinical signs and symptoms to indicate biofilm presence in chronic wounds in the absence of practical and convenient diagnostic tests [23], but we have not encountered extensive attempts to validate them or collate those reported in the literature into a definitive list.

When thinking about treating or managing biofilm, we ask that if there is uncertainty around its diagnosis and we are relying on unvalidated criteria for confirmatory purposes at the bedside, how do we know that it has truly been removed, and can we be positive that it was there in the first place?

To our knowledge, a structured review to map the literature and collate a comprehensive list of signs and symptoms currently used to indicate the presence of biofilm in chronic wounds has not been undertaken. This review is not an attempt to determine that the signs and symptoms we encounter are those we should be seeking to confirm biofilm presence, rather it is an attempt to address a gap by identifying and cataloguing which signs, symptoms and/or biomarkers within the literature are reported to indicate the presence of biofilm in chronic wounds.

## 2 Materials and Methods

### 2.1 Design

A scoping review methodology was used to identify signs and symptoms reported in the literature to be indicative of biofilm in chronic wounds. The Preferred Reporting Items for Systematic Reviews and Meta‐Analyses extension for Scoping Reviews (PRISMA‐ScR) statement and the Joanna Briggs Institute Manual for Evidence Synthesis guided the work [28, 29].

A protocol for this review has been published on the HRB Open Research Website [30], and the key stages are summarised below.

### 2.2 Search Strategy and Information Sources

Using keywords and controlled vocabulary identified from articles in the authors' possession and from articles retrieved through preliminary searches of Ovid Medline and EBSCO CINAHL, a search strategy was developed in Ovid Medline [30], peer reviewed. according to Peer Review of Electronic Search Strategies (PRESS) guidelines [31], and run in Ovid Medline, Ovid Medline Daily, Ovid Medline In‐Process and Other Non‐Indexed Citations, Ovid Medline ePub Ahead of Print, Embase, CINAHL and Cochrane CENTRAL. The Bielefeld Academic Search Engine (BASE) was searched for relevant grey literature. The literature search was completed on July 06th 2023. No limits were applied, and all databases were searched from inception to the date on which searches were run.

Reference lists of a random sample of 10% of eligible reports included in the review were scanned, contributions were made by a content expert and authors of included material were contacted to provide more information where necessary.

### 2.3 Eligibility Criteria

#### 2.3.1 Inclusion Criteria

Articles considered eligible for this review included but were not limited to systematic reviews, randomised controlled trials (RCTs), controlled clinical trials, cohort studies, case‐controlled studies, case series and case reports. Also deemed eligible were letters to the editor with relevant data and editorials.

Articles had to include or refer to adult patients (18+ years) with venous leg ulcers (VLU), diabetic foot ulcers (DFU), pressure ulcers (PU) and/or mixed arterial/venous leg ulcers (MAVLU) treated in any setting, and report on clinical signs, symptoms and/or biomarkers validated or otherwise thought to be associated with the presence of biofilm in chronic wounds.

#### 2.3.2 Exclusion Criteria

Articles exclusively including or referring to patients with wounds resulting from burns, malignant fungating wounds, wounds secondary to conditions such as rheumatoid arthritis or pyoderma gangrenosum. These wounds have a unique aetiology and physiology that differentiates them from the ‘typical’ wound, for example VLU, DFU, PU, MAVLU and as such they need to be managed differently.

### 2.4 Selection of Evidence

Search results were exported to EndNote X9, deduplicated and then transferred to the Rayyan on‐line screening tool [32], where they were deduplicated for a second time.

#### 2.4.1 Level 1 Screening (Title and Abstract Screening)

A pilot screening exercise was conducted on a sample of 50 titles and abstracts. A follow‐up meeting to resolve discrepancies was held and following the meeting all reviewers voted to proceed with the full screening exercise.

Reviewer pairs (J.D.I. & G.G.; J.D.I. & L.P.; J.D.I. & A.V.; J.D.I. & P.C.; J.D.I. & D.S.; J.D.I. & C.H.; J.D.I. & C.F.) independently screened titles and abstracts for inclusion into the review against the pre‐determined eligibility criteria. A single failed criterion was considered sufficient to exclude a study. Discrepancies were resolved by discussion between authors in a pair with referral to a third party for a final decision where necessary.

#### 2.4.2 Level 2 Screening (Full Text Screening)

Full text articles of titles and abstracts retained after title and abstract screening were located by JDI with additional assistance from the University of Galway's James Hardiman Library Interlibrary Loan Service where necessary.

A pilot screening exercise was conducted on a sample of 10 full text articles. A follow‐up meeting was held to resolve discrepancies and following the meeting all reviewers voted to proceed with the screening exercise proper.

Pairs of authors (J.D.I. & G.G.; J.D.I. & L.P.; J.D.I. & A.V.; J.D.I. & C.H.; J.D.I. & D.S.; J.D.I. & C.F.) independently screened full text articles against the pre‐determined eligibility criteria. Again, an article could be excluded based on a single failed eligibility criterion. Discrepancies were resolved by discussion between authors in a pair with referral to a third party where necessary.

Full text articles located through reference scanning were screened for eligibility by a single review author (JDI).

### 2.5 Data Charting Process and Data Items

A data extraction form was developed a priori in Microsoft Excel (2016). The form was designed to capture information including but not limited to study characteristics (author, year, study design/article type, country of origin), patient characteristics (age, sex, ethnicity), wound characteristics (aetiology, dimensions, and duration) and reported signs and symptoms/biomarkers of biofilm in chronic wounds.

Data was extracted by a single study author (JDI) and the extracted data from 21 (19%) randomly chosen included articles was verified by a second party (RJ). In the event of a discrepancy, resolution required discussion between both parties with the option to defer to the opinion of a third party if necessary.

For research articles, for example randomised controlled trials or cohort studies, eligible concept data was only extracted from methods and results sections, for other article types, for example literature reviews or editorials, concept data could be extracted from any section.

Characteristics of included studies were presented in tabular form, and graphically or textually as frequency of included reports by publication year, country of origin, study design/article type, wound aetiology and setting.

Extracted concept data (signs/symptoms reported in the literature to be indicative of biofilm in chronic wounds) was reviewed by study authors and categorised using an inductive content analysis methodology.

The categorised concept data was presented graphically and in tabular form as frequency counts in terms of overall occurrence and occurrence by study design/article type.

Descriptive statistics (mean, standard deviation (SD) and median, interquartile range (IQR) and range) were incorporated with concept data where appropriate.

## 3 Results

### 3.1 Search Results

**FIGURE 1:**
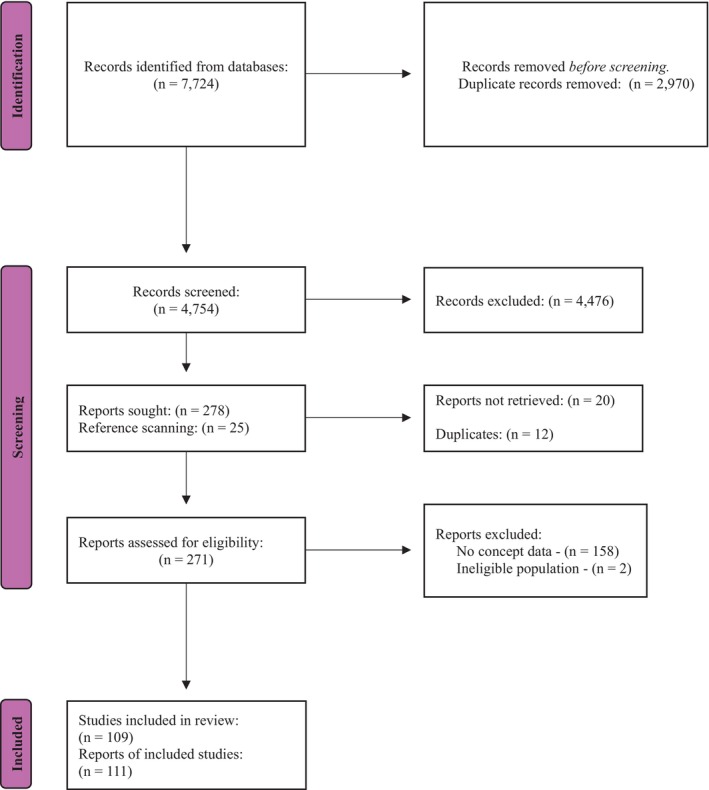
PRISMA flow diagram of search results (inception to July 6th 2023) [47].

The search strategy returned 7724 records. Post deduplication, 4754 titles and abstracts were screened for eligibility (Figure 1). Two‐hundred and seventy‐eight were passed up for full‐text screening and of these, 12 were duplicates, 16 were irretrievable and 148 failed eligibility criteria (provided no concept data [reported signs and/or symptoms of biofilm in chronic wounds] or had an ineligible population) (Appendix B: Table of excluded studies).

Reference scanning and contributions from content experts provided 25 full text articles (Figure 1). Four of these were irretrievable and 12 did not meet the review eligibility criteria (provided no concept data or had an ineligible population) (Appendix B: Table of excluded studies).

### 3.2 Characteristics of Included Studies

One‐hundred and eleven reports of 109 primary articles were included in this review (Appendix A: Characteristics of included studies).

**FIGURE 2:**
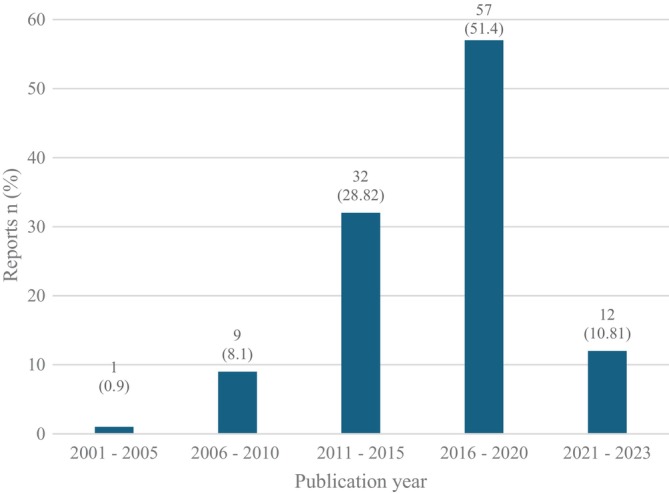
Included reports *n* (%) by year of publication.

The majority of eligible reports were published between 2011 and 2020 while almost none appeared prior to 2006 (Figure 2).

More than 20 countries were represented with 41 (37%) reports originating from the United Kingdom (UK) followed by 14 (12.6%) from Australia, 14 (12.6%) from the United States of America (USA) and six (5.4%) coming from Spain. Canada, Portugal, China, Colombia, Brazil, Denmark, Germany and Italy provided between two (1.8%) and five (4.5%) reports each, while Austria, Belgium, India, Ireland, Japan, Mexico, The Netherlands, Poland, South Africa, Switzerland and Taiwan each provided one (0.9%) report.

**FIGURE 3:**
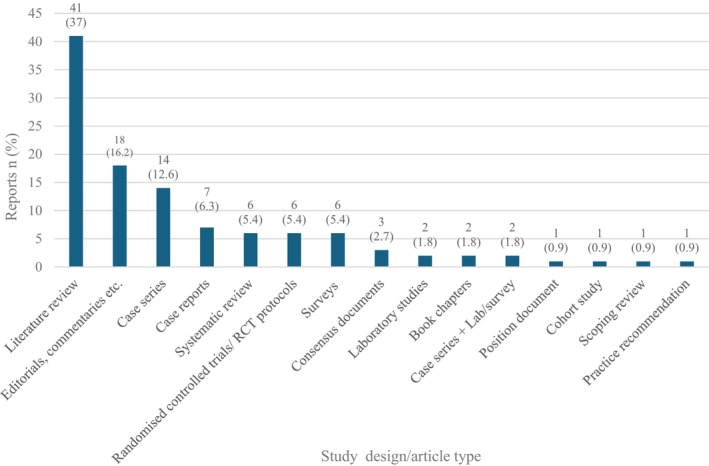
Included reports *n* (%) by study design/article type.

In terms of study design/report type, the majority were classed as literature review type articles with no elements of systematic review methodology reported. Two of these had an associated case series and one an associated case report. The least common report types were position documents, practice recommendations, cohort studies and scoping reviews of which one each (0.9%) were included (Figure 3).

The setting was recorded in 31 (28%) reports. Dedicated wound care clinics were the setting of choice in 11 (10%) reports followed by a virtual setting in 6 (5.41%). Five (4.5%) studies took place in a hospital setting and two (1.8%) recorded a routine care setting/public health centre. Seven (6.31%) took place in undefined healthcare facilities or specified hospital departments. Setting was not recorded or did not apply in 80 (72%) reports.

**FIGURE 4:**
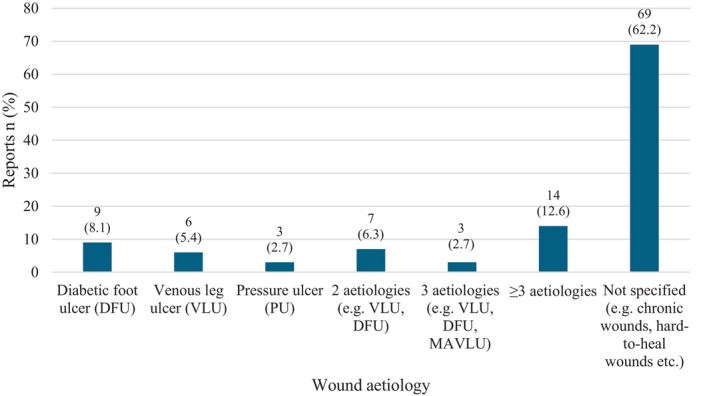
Included reports *n* (%) by wound aetiology.

Diabetic foot ulcers were the most common aetiology. Sixty‐nine (62.2%) reports did not specify an aetiology and typically made reference to “chronic wounds”, “complex wounds”, “hard‐to‐heal wounds” and so forth (Figure 4). Patient and wound demographic data were recorded inconsistently and appeared in 24% and 16% respectively of the included reports. This data is not representative of the whole sample of included articles and is not presented in this review.

### 3.3 Reported Signs and Symptoms of Biofilm in Chronic Wounds

Signs and symptoms reported in the literature as being indicative of biofilm were encountered 830 times in the 111 included reports. The average number of signs and symptoms per included report was 7.4, SD 8.8 (median five, IQR five). The range was from 1 to 51.

**FIGURE 5:**
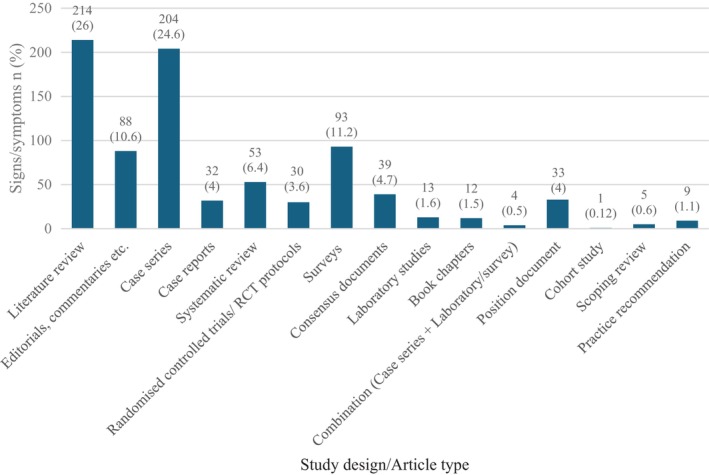
Signs and symptoms reported to be indicative of biofilm in chronic wounds *n* (%) by study design/article type.

In terms of study design, literature reviews produced 214 (26%), case series produced 204 (24.6%) and surveys produced 93 (11.2%) reported signs and symptoms. The least number of signs and symptoms produced was one (0.12%) and this came from a cohort study design (Figure 5).

Within the included articles, signs and symptoms were reported in different ways using different descriptions and terminologies. References to delayed healing included for example “Indolent wound despite healing”, “wound is not healing”, “delayed healing” or “stalled wound”, and references to exudate included “copious exudate”, “exudating”, or “excessive exudate”. Following discussion and review among co‐authors they were grouped into a series of 26 more comprehensive and succinct sign/symptom statements (Table 1). Visual indicators were reported most frequently and were encountered 198 (24%) times followed by failure to respond as expected to antimicrobial therapies which occurred 125 (15%) times and failure of a wound to progress to healing which occurred 108 (13%) times. Wound duration of more than 6 weeks and extreme tolerance to host defences were encountered only one time (0.9%) each across all included articles.

**TABLE 1:** Refined and categorised signs and symptoms reported in the literature to be indicative of biofilm in chronic wounds.

	Sign/symptom	Occurrences in literature *n* (%)
1	Visual indicators: A shiny, slimy layer on a non‐healing wound surface that persists despite autolytic or enzymatic debridement but can be removed easily with physical removal techniques such as cleansing or mechanical debridement. The surface reforms quickly (1–2 days) in the absence of frequent intervention. For example, cleansing, debridement.	198 (24)
2	Failure to respond as expected to antimicrobial therapies. For example, systemic antibiotics and/or topical antimicrobials/antiseptics. Infection may re‐emerge when treatment is completed.	125 (15.1)
3	Wound fails to close or progress to healing despite optimal wound/patient management.	108 (13.01)
4	Increased exudate.	63 (7.6)
5	Presence of slough or fibrin.	50 (6)
6	Signs of local infection. For example, erythema, oedema, serous effusion, new areas of breakdown, pain etc.	45 (5.42)
7	Persistent or prolonged (low‐level) inflammation.	43 (5.2)
8	Poor quality granulation tissue. For example, discoloured, friable, fragile or hypergranulation.	42 (5.1)
9	Signs of necrotic tissue/eschar.	28 (3.4)
10	Infection lasting more than 30 days or persistent cycles of recurrence (waxing and waning).	23 (2.8)
11	Favourable response to therapies such as corticosteroids, tumour necrosis factor (TNF) α inhibitors, or multimodal strategies such as physical debridement, cleansing and topical antimicrobial agents and dressings.	21 (2.5)
12	Malodour.	20 (2.41)
13	Negative cultures despite signs/suspicion of clinical infection.	15 (1.81)
14	Wound pain/pain at site.	12 (1.5)
15	Low‐level/increased erythema.	10 (1.2)
16	Wound edge. For example, maceration, non‐physiological colour changes of the wound margin, hyperkeratosis.	4 (0.5)
17	Swelling/wound bed with pale oedema.	4 (0.5)
18	Debris or foreign objects in the wound. For example, pins, mesh, prosthetic devices.	4 (0.5)
19	Soft tissue deterioration despite antibiotic treatment and/or surgical debridement.	4 (0.5)
20	Tunnelling (along tissue structures, degradation along solid structures such as tendon or bone) and/or undermining (erosion of the subcutaneous layer under the wound edge).	3 (0.4)
21	Temperature. Sometimes low‐grade fever.	2 (0.24)
22	History of systemic antibiotics in the last 12 months, prior or current use of antimicrobials.	2 (0.24)
23	Wound duration of more than 6 weeks.	1 (0.12)
24	Extreme tolerance to host immune defence.	1 (0.12)
25	Sepsis.	1 (0.12)
26	Other patient history indicators. For example, change in the patient's overall wellbeing or quality of life.	1 (0.12)

## 4 Discussion

Currently, there are no clear‐cut or routine tests available for use at the bedside to help clinicians determine if biofilm is present in chronic wounds [23, 25]. A study published in September of this year involving 40 patients with chronic wounds demonstrated that a fluorescence imaging device and a wound blotting technique had sensitivities of 84% and 24% respectively, specificities of 27% and 64%, and accuracies of 63% and 40% for detecting biofilm when compared against scanning electron microscopy (SEM) and polymerase chain reaction (PCR) microbiology gold standards [33]. The most reliable approaches include advanced microscopy techniques that are not intended for use in bedside practice [23, 24, 34]. Overt or classic (traditionally associated with local infection) signs and symptoms such as erythema, warmth, swelling and malodour, or covert (subtle, typically manifesting before the onset of overt indications) signs and symptoms such as hypergranulation, increasing exudate and delayed healing are routinely used to diagnose infections in chronic wounds [35], and given the diagnostic issues described above it is not surprising that a similar approach would be applied to determining presence of biofilm. Indeed, the statement “In the absence of bedside diagnostic tests, specific signs and symptoms should be used to confirm biofilm presence” received strong agreement in a 2017 consensus guideline although it was not a key statement [23].

This scoping review included 111 reports that yielded 830 accounts of a clinical sign or symptom thought to be indicative of biofilm in chronic wounds. Some reports provide a mixture of visual cues and other clinical signs. Metcalf et al. for example refer to “viscous, slimy or gel‐like substances that can form on, beneath or even within some dressings”, excessive moisture and poor quality granulation tissue [36]. Bowlby & Blume write that “the presence of slough, shininess to an ulcer, malodour or necrotic tissue is likely an indication that biofilm is present” [37]. Other authors tend to emphasise the visual. Hurlow et al. describe a “cloudy, shiny, thin slime material on the ulcer bed that persisted despite daily cleaning with pulsed lavage”, and “a cloudy, translucent film on the wound surface through which larger granular buds protruded” in their 2009 case series [38]. Still others describe only clinical cues. Haesler and Ousey write that chronic wounds exhibiting delayed healing despite optimal conditions, recalcitrance to antimicrobial therapy, and antibiotic failure may have biofilm present. In addition, they refer to signs of early local infection such as increasing exudate and moisture, low level inflammation and erythema, and poor granulation or friable hypergranulation [39].

However, while it is important to recognise symptoms of wound biofilm, clinical diagnosis can be subjective [40]. it is also acknowledged that wound biofilms are difficult to visualise macroscopically, with the possibility of mistaking slough, debris and exudate for biofilm [23]. In addition, slough in a wound and other visual markers of biofilm have been deemed problematic and seen as being too subjective, without basis and objective criteria [40]. Interestingly, visual cues were the most encountered sign of biofilm in this review. The frequency of reporting of a sign or symptom thought to be indicative of biofilm does not necessarily reflect its significance or validity, that is, a sign or symptom reported 20 times holds no more importance in terms of validity than one reported two or three times. Indeed, of the included reports in this review, only two provided validation data in relation to signs and symptoms of biofilm in chronic wounds [41, 42]. Using histology (haematoxylin and eosin staining) and SEM, Hurlow et al. identified biofilm in 12/16 (75%) of samples of a “firmly adherent, gel‐like wound bed film which had reformed under antiseptic wound dressings and sometimes despite guided systemic antibiotics” taken from 11 consenting participants with chronic non‐healing wounds [41].

As a secondary objective in their microscopy visualisation study, Johani et al. [42] set out to correlate six clinical wound observations (presence of slough, excess exudate, poor tissue quality, signs of pyocyanin, gelatinous wound surface and gelatin reforming quickly) against microscopy (peptide nucleic acid fluorescent in situ hybridisation [PNA‐FISH] confocal laser scanning microscopy [CLSM]) of biopsies taken from 26 diabetic foot ulcers. They found that apart from excessive exudate, the probability of them facilitating clinicians in identifying wound biofilm was no better than chance alone. They proposed that clinical cues are not useful for detecting biofilm presence in these ulcers, but that larger sample sizes and other chronic wound aetiologies would be required to verify their results. Subsequent to this review, a study from 2024 reported the sensitivity, specificity and accuracy of clinical signs of biofilm to be 44%, 40% and 43% respectively in chronic wounds when compared against SEM and PCR microbiology gold standards. However, it is not clear from the study how these signs were assessed, that is, as single entities or as aggregates/combinations [33].

Solutions to the biofilm diagnosis problem in chronic wounds could perhaps take the form of making microscopy methodologies a more routine part of clinical/laboratory practice. More realistically, we could develop bedside diagnostic devices, indeed there are novel technologies currently in existence such as wound blotting and point‐of‐care fluorescence detection [33, 43, 44]. Another avenue could be to work with clinical indicators and seek to further determine their diagnostic capabilities through validation work as has been done in the case of acute foot and ankle injuries and systemic lupus erythematosus (SLE) [45, 46].

### 4.1 Limitations

Due to time and resource restrictions, rather than have two reviewers independently extract data, one reviewer extracted data, and this was verified in 21 (19%) of the included articles by a second party. However, the sample for verification was chosen randomly and the agreement rate in that sample was almost 100%.

Reference scanning returned nine eligible full texts, and it is possible that had the reference lists of all included articles been scanned rather than a percentage, more eligible articles could have been returned. Nevertheless, given that the purpose of this review was to collate a list of signs and symptoms reported in the literature to be indicative of biofilm in chronic wounds, and that extracted data was repetitious in nature with the same signs and symptoms appearing time and time again, it is unlikely that any new or unexpected data would have been retrieved. For the same reasons it was thought unlikely that extensive efforts to contact content experts would add anything new to the review. This review was concerned more with producing a comprehensive list of signs and symptoms than with documenting the exact number reported in the literature and so the methodology as it stands was adequate to achieve this.

Finally, the topic of interest for this review is not typically reported as primary or secondary outcomes data that would appear in an article abstract and so it is possible that eligible articles were missed in the title and abstract screening phase. It would not have been feasible to locate and screen full texts of all titles and abstracts, but our search strategy was comprehensive. Four literature databases and one grey literature source were searched with an extensive strategy from inception to date of search without any limits, and the search was subsequently updated. In addition, as stated above, observing the same signs and symptoms appear repeatedly lead us to conclude that no significant data was missed.

## 5 Conclusion

It would seem apparent that in terms of using visual cues and clinical signs and symptoms to determine presence of biofilm in chronic wounds, the territory is not very clearly mapped out. The concept is commonly recommended and employed but is not without its critics. This review recovered numerous accounts of signs and symptoms which we refined into a series of statements deemed to be indicative of biofilm in chronic wounds, but it only uncovered validation work on four of these (presence of exudate, slough, poor tissue quality and a quickly returning gelatinous surface), the results of which are not particularly supportive of the idea.

It could be argued that confirming presence of biofilm in chronic wounds is not necessary, that we are over‐complicating matters and that it would be more beneficial in terms of patient outcomes and resource use were we just to focus on debriding away devitalised tissue, biofilm and other debris to promote wound healing. However, it is generally accepted that biofilm contributes to delayed healing, and we are unaware of any new evidence that would ask us to reconsider that consensus. Indeed, a commonly reported indicator in the literature refers to delayed healing *despite optimal management*. Furthermore. global expenditure on biofilm‐related complications in chronic wounds is high and there are many treatment strategies that claim to completely remove biofilm, but their supporting evidence largely comes from in vitro work which demonstrates efficacy but not necessarily real‐life effectiveness.

We would therefore advocate that in the interests of optimising patient care and streamlining treatment strategies, a concerted effort to validate or nullify the reported signs and symptoms of biofilm in the literature be carried out and the findings of this scoping review could serve as an ideal starting point.

## Conflicts of Interest

The authors declare no conflicts of interest.

## Supporting information


Data S1.

## Data Availability

The data that support the findings of this study are available from the corresponding author upon reasonable request.

## Appendix A

Characteristics of included studies.

**Table:**

Reference	Year of publication	Country of origin	Study design	Setting	Wound aetiology
Afonso AC, Oliveira D, Saavedra MJ, Borges A, Simoes M. Biofilms in Diabetic Foot Ulcers: Impact, Risk Factors and Control Strategies. International Journal of Molecular Sciences. 2021;22 (15):25.	2021	Portugal	Literature review, non‐systematic	Not applicable	Diabetic foot ulcers
Aswathanarayan JB, Rao P, Hm S, Gs S, Rai RV. Biofilm‐Associated Infections in Chronic Wounds and Their Management. Advances in Experimental Medicine and Biology. 2023;1370:55–75.	2023	India	Literature review, non‐systematic	Not applicable	Chronic wounds (including vascular, diabetic, pressure and burns)
Bartoszewicz M, Junka A. Biofilm based wound care: strategy for the treatment of chronic wounds affected by the infection caused by microorganisms in the form of biofilms. Leczenie Ran. 2012;9 (1):1–6.	2012	Poland	Literature review, non‐ systematic	Not applicable	Chronic wounds
Becerra G CC, García A MP, Reyes M YD, Huertas MG. Bacterial biofilms in chronic wounds; Biopeliculas bacterianas em feridas crónicas; Biopelículas bacterianas en heridas crónicas. Rev. Health Forest. 2019;9 (1):47–61.	2019	Colombia	Literature review, non‐systematic	Not applicable	Chronic wounds
Beele H, Van Overschelde P, Olivecrona C, Smet S. A prospective randomised controlled clinical investigation comparing two post‐operative wound dressings used after elective hip and knee replacement; Mepilex R Border Post‐Op versus Aquacel R surgical. International Journal of Orthopaedic and Trauma Nursing. 2020;38:100772.	2010	Belgium	Randomised controlled trial	Not reported	Pressure ulcers, Venous leg ulcers
Bernardo Martín A. Eficacia del cadexómero yodado frente a la hidrofibra de hidrocoloide con plata combinado con desbridamiento cortante en el tratamiento de úlceras por presión con sospecha de biofilm bacteriano: Ensayo clínico con diseño factorial; Efficacy of iodine cadexomer versus hydrocolloid hidrofyber with silver in pressure ulcers treatment with bacterial biofilm suspicion: Factorial design clinical trial. Santander. Universidad de Cantabria; 2017.	2017	Spain	Randomised controlled trial protocol (Thesis)	Multicentre, public, subsidised, and private socio‐sanitary centres, urban primary care centres	Pressure ulcers
Bjarnsholt T, Alhede M, Jensen PO, Nielsen AK, Johansen HK, Homøe P, et al. Antibiofilm properties of acetic acid. Adv Wound Care. 2015;4 (7):363–72.	2015	Denmark	Case series + Laboratory	Not reported	Diabetic foot ulcers, Leg ulcers
Bjarnsholt T, Kirketerp‐Møller K, Jensen PØ, Madsen KG, Phipps RK, Krogfelt K, et al. Why chronic wounds will not heal: a novel hypothesis. Wound Repair & Regeneration. 200 816 (1): 2–10.	2008	Denmark	Literature review, non‐systematic (hypothesis paper/perspective article)	Not applicable	Venous leg ulcers, Pressure ulcers, Diabetic foot ulcers
Bowen G, Richardson N. Biofilm management in chronic wounds and diabetic foot ulcers. Diabetic Foot Journal. 2016;19 (4):198–204.	2016	UK	Literature review, non‐systematic	Not applicable	Diabetic foot ulcers, Leg ulcers
Bowlby M, Blume P. Can ultrasound debridement facilitate biofilm removal from diabetic foot ulcers? Podiatry today. 2014;27 (8):20–9.	2014	USA	Literature review, non‐systematic	Not applicable	Diabetic foot ulcers
Brill FHH, Wodrich M, Pahl S. Biofilms in chronic wounds: Emergence and clinical relevance for wound healing. Vasomed. 2017;29 (4):172–3.	2017	Germany	Editorial (summary of a congress presentation)	Not applicable	Chronic wounds
Brown A. Diagnosing and managing wound infection. Journal of Community Nursing. 2022;36 (4):26–34.	2022	UK	Literature review, non‐systematic	Not applicable	Chronic, acute, surgical wounds
Brown A. Dispelling some myths and misconceptions in wound care. Journal of Community Nursing. 2018;32 (6):24–32.	2018	UK	Literature review, non‐systematic	Not applicable	Wounds
Castrillón Rivera LE, Palma Ramos A, del Carmen Padilla Desgarennes M. Interferencia de las biopelículas en el proceso de curación de heridas. Dermatología Rev. Mex. 2011;55 (3):127–39.	2011	Mexico	Literature review, non‐systematic	Not applicable	Chronic wounds
Choudhury M, Downie F. A biofilm based wound care pathway in the community setting: a review. Wounds UK. 2022;18 (4):14–20.	2022	UK	Literature review, non‐systematic	Not applicable	Chronic, non‐healing wounds
Cogo A, Bignozzi AC, Hermans MH, Quint BJ, Snels JP, Schultz G. A desiccation compound as a biofilm‐ and necrosis‐removing agent: a case series. Journal of Wound Care. 2022;31 (10):816–22.	2022	Italy	Case series	Hospital	Venous leg ulcers, Post‐traumatic, Vascular ulcers, Ischaemic ulcers, Diabetic foot ulcers
Cooper R. THE ROLE OF BIOFILMS IN CHRONIC FOOT WOUNDS. Podiatry Now. 2016;19 (11):30–1.	2016	UK	Literature review, non‐systematic	Not applicable	Diabetic foot ulcers
Cowan T. Biofilms and their management: from concept to clinical reality. Journal of Wound Care. 2011;20 (5):220–6.	2011	UK	Editorial (Report on a lecture)	Not applicable	Chronic wounds
Cowan T. Prevention and management of wound biofilms: what are the options? A round table discussion. Journal of Wound Care. 2011;20 (5):227–30.	2011	UK	Editorial (Report on a round table discussion)	Not applicable	Wounds
Cowan T. Visible biofilms—a controversial issue! Journal of Wound Care. 2012;21 (3):106.	2012	UK	Editorial	Not applicable	Wounds
Cowan T. Biofilms and their management: implications for the future of wound care. Journal of Wound Care. 2010;19 (3):117–20.	2010	UK	Editorial (lecture report)	Not applicable	Chronic wounds
Cutting K, Wolcott R, Dowd S. Biofilms and Significance to Wound Healing. In: Percival S & Cutting K., editors. Microbiology of Wounds: CRC Press; 2010. p. 233–47.	2010	UK	Book Chapter	Not applicable	Wounds
Darvishi S, Tavakoli S, Kharaziha M, Girault HH, Kaminski CF, Mela I. Advances in the Sensing and Treatment of Wound Biofilms. Angewandte Chemie (International ed. in English). 2022; 61 (13): e202112218	2021	Switzerland	Literature review, non‐systematic	Not applicable	Chronic wounds
Dawkins H. Non‐healing venous leg ulcer. British Journal of Nursing. 2017;20:S26‐S7.	2017	UK	Case report	Not reported	Venous leg ulcers
de Moura MRL, Soares SR, de Azevedo DS, Miranda JS. Treatment protocol for hard‐to‐heal wounds using a hydrofiber dressing with 1.2% ionic silver ethylenediaminetetraacetic acid and benzethonium chloride. Journal of Wound Care. 2020;29 (10):18–26.	2020	Brazil	Case series	Outpatient centre caring for patients referred from the municipal health department for diagnosis and treatment in the areas of angiology and vascular surgery.	Diabetic foot ulcers, Venous leg ulcers
Dowsett C. Biofilms: A practice‐based approach to identification and treatment. Wounds UK. 2013;9 (2):68–72.	2013	UK	Literature review, non‐systematic (Practice development)	Not applicable	Chronic wounds
Dowsett C, Bellingeri A, Carville K, Garten A, Woo K. A route to more effective infection management: The Infection Management Pathway. Wounds International. 2020;11 (3):50–7.	2020	UK	Survey + report of an infection management pathway	Australia, New Zealand, Europe, North America	Diabetic foot ulcers, Venous leg ulcers, Pressure ulcers, Arterial ulcers & Surgical dehisced wounds
Drago F, Gariazzo L, Cioni M, Trave I, Parodi A. The microbiome and its relevance in complex wounds. Eur J Dermatol. 2019;29 (1):6–13.	2019	Italy	Literature review, non‐systematic + case series	Hospital ulcer clinic	Pressure ulcers, diabetic foot ulcers
Durbin K. A three‐patient evaluation. British Journal of Nursing (Mark Allen Publishing). 2017;26 (Sup20a):S21‐S2.	2017	UK	Case series	Acute hospital	Venous leg ulcers, unknown aetiology
Edwards‐Jones VAL, Atkin L, Guttormsen K. Biofilm‐based wound care: how to cleanse, debride and manage chronic wounds. Wounds UK. 2018;14 (4):10–6.	2018	UK	Editorial (expert debate)	Not applicable	Chronic wounds
Gagnon L. Biofilm presents challenge in wound care. Dermatology Times. 2015;36 (1):20.	2015	Canada	Editorial	Not applicable	Wounds
Geng F, Cao S, Bo S, Feng M, Jia Y, Quin J. Effect and cost ⁃ effectiveness analysis on using two antibacterial silver dressings on infection of wound with bacterial biofilm. Chinese Nursing Research. 2022;36 (1):44–8.	2022	China	Randomised controlled trial	Wound clinic of a tertiary grade hospital	Trauma, Burns, Venous leg ulcers, Surgical wounds, Pressure ulcers, diabetic foot ulcers
Gillies A. Surfactants in the treatment of chronic wounds and biofilm management. Journal of Community Nursing. 2019;33 (6):34–8.	2019	UK	Literature review, non‐systematic	Not applicable	Chronic wounds
Gompelman M, van Asten SAV, Peters EJG. Update on the Role of Infection and Biofilms in Wound Healing: Pathophysiology and Treatment. Plastic and Reconstructive Surgery. 2016;138 (3 Suppl):61S‐70S.	2016	USA	Literature review, non‐systematic	Not applicable	Diabetic foot ulcers
Haemmerle G, Duelli H, Abel M, Strohal R. The wound debrider: A new monofilament fibre technology. British Journal of Nursing. 2011;20 (6 SUPPL.):S35‐S42.	2011	Austria	Case series	2 hospitals	Diabetic foot ulcers, Venous leg ulcers, Arterial ulcers, Mixed arterial/venous leg ulcers
Haesler E, Ousey K. Evolution of the wound infection continuum. Wounds International. 2018;9 (4):6–10.	2018	Australia	Editorial	Not applicable	Wounds
Haesler E, Swanson T, Ousey K, Carville K. Clinical indicators of wound infection and biofilm: reaching international consensus. Journal of Wound Care. 2019;28 (3):S4‐S12.	2019	Australia	eDelphi Survey	Online	Chronic wounds
Hampton S. Managing biofilm by using dressings. British Journal of Community nursing. 2015;Community Wound Care:S10, S2.	2015	UK	Editorial (Clinical comment)	Not applicable	Chronic wounds
Hernández Ortiz JÁ, Navarro Fernández AM, Galera Barrero AM, Marín Bello MJ, Cazalilla Pérez A, Lopes Pereira AM. Abordaje de una herida por roce o fricción de categoría III, con presencia de biofilm, tras contención mecánica. Gerokomos. 2018;29 (2):105–7.	2018	Spain	Case report	Intensive care unit	Pressure ulcers
Hsiao‐Chen L. Using aquaceltm ag + dressing in treating poor healing chronic wound after surgical debridement—Case report and literature review. Journal of Wound Care. 2020;29 (SUPPL 7B):217.	2020	Taiwan	Case report	Surgical department	Chronic wounds
Hurlow J, Bowler PG. Potential implications of biofilm in chronic wounds: a case series. Journal of Wound Care. 2012;21 (3):109–10, 12, 14 passim.	2012	USA	Case series	Acute and outpatient	Venous leg ulcers, Leg ulcers, Pressure ulcers, Mixed Arterial Venous leg ulcers, traumatic, oedema, burns
Hurlow J, Bowler PG. Clinical experience with wound biofilm and management: A case series. Ostomy Wound Management. 2009;55 (4):38–49.	2009	USA	Case series	Not reported	Venous leg ulcers, Ischaemic ulcers
Hurlow J. Understanding biofilm: what a community nurse should know. British Journal of Community Nursing. 2016;21:S26‐S33.	2016	UK	Literature review, non‐systematic	Not applicable	Chronic wounds, Venous leg ulcers
Hurlow J, Blanz E, Gaddy JA. Clinical investigation of biofilm in non‐healing wounds by high resolution microscopy techniques. Journal of Wound Care. 2016;25:S11‐S22.	2016	USA	Laboratory study	Wound care centre	Leg ulcers, Venous leg ulcers, traumatic, Ischaemic ulcers
Johani K, Malone M, Jensen SO, Gosbell IB, Dickson HG, Hu H, et al. Microscopy visualisation confirms multi‐species biofilms are ubiquitous in diabetic foot ulcers. International Wound Journal. 2017;14 (6):1160–1169	2017	Australia	Laboratory study	High risk foot service	Diabetic foot ulcers
Kataoka Y, Kunimitsu M, Nakagami G, Koudounas S, Weller CD, Sanada H. Effectiveness of ultrasonic debridement on reduction of bacteria and biofilm in patients with chronic wounds: A scoping review. International Wound Journal. 2021;18 (2):176–86.	2020	Japan	Scoping review	Not applicable	Venous leg ulcers
Keast D, Swanson T. Clinical innovation: 2016 wound infection consensus document. Wounds International. 2017;8 (2):19–21.	2017	Australia	Editorial	Not applicable	Wounds
Swanson T, Keast D, Cooper R, Black J, Angel D, Schultz G, et al. Ten top tips: identification of wound infection in a chronic wound. Wounds International. 2015;6 (2):22–7.	2014	Canada	Literature review, non‐systematic	Not applicable	Chronic wounds
Leaper DJ, Schultz G, Carville K, Fletcher J, Swanson T, Drake R. Extending the TIME concept: What have we learned in the past 10 years? International Wound Journal. 2012;9 (SUPPL. 2):1–19.	2012	UK	Literature review, non‐systematic	Not applicable	Chronic wounds
Lenselink E, Andriessen A. A cohort study on the efficacy of a polyhexanide‐containing biocellulose dressing in the treatment of biofilms in wounds. Journal of Wound Care. 2011;20 (11):534–9.	2011	Netherlands	Cohort	Outpatient wound clinic	Venous leg ulcers, Mixed arterial/venous leg ulcers, Surgical wounds, Burns, Arterial ulcers, Diabetic foot ulcers
Lloyd Jones M. Series 3. Wound infection 3.1: the wound infection continuum explained. British Journal of Healthcare Assistants. 2018;12 (5):218–20.	2018	UK	Editorial (review of a best practice statement)	Not applicable	Wounds
Malone M, Schultz G. Challenges in the diagnosis and management of wound infection. The British Journal of Dermatology. 2022;187 (2):159–66.	2022	Australia	Literature review, non‐systematic	Not applicable	Multiple aetiologies including but not limited to diabetes‐related foot ulcers, venous leg ulcers, surgical wounds, and burns.
Malone M, Schwarzer S, Radzieta M, Jeffries TT, Walsh A, Dickson HG, et al. Effect on total microbial load and community composition with two vs. six‐week topical Cadexomer Iodine for treating chronic biofilm infections in diabetic foot ulcers. International Wound Journal. 2019;16 (6):1477–86.	2019	Australia	Randomised controlled trial	Acute tertiary referral hospital	Diabetic foot ulcer
McGuire J, D'Alessandro J. Combating Biofilms In The Chronic Wound. Podiatry Today. 2016;29 (8):32–40.	2016	USA	Literature review, non‐systematic	Not applicable	Chronic wounds
McGuire J, Love E, Vlahovic TC, Khan K, Labbad ZG, Robinson L, et al. The ABCESS System for Chronic Wound Management: A New Acronym for Lower Extremity Wound Management. Wounds: a Compendium of Clinical Research and Practice. 2020;32:S1‐S25.	2020	USA	Literature review, non‐systematic (Acronym proposal)	Not applicable	Chronic wounds/lower extremity wounds
Mendoza RA, Hsieh J‐C, Galiano RD. The Impact of Biofilm Formation on Wound Healing. In: Hakan Dokan K, editor. Wound healing—Current Perspectives: IntechOpen; 2019. p. 235–50.	2019	USA	Book chapter	Not applicable	Chronic wounds
Metcalf D, Bowler PG. Perceptions of Wound Biofilm by Wound Care Clinicians. Wounds‐Compend Clin Res Pract. 2019;31 (3):E14‐E7.	2019	UK	Survey	Online (UK, Germany, USA, Italy)	Non‐healing wounds
Metcalf DG, Bowler PG. Biofilm delays wound healing: A review of the evidence. Burns Trauma. 2013;1 (1):5–12.	2013	UK	Literature review, non‐systematic	Not applicable	Chronic wounds, Venous leg ulcers, Diabetic foot ulcer + Cellulitis, Peripheral arterial ulcers, highly exuding ulcer
Metcalf DG, Bowler PG. Clinician perceptions of wound biofilm. International Wound Journal. 2016;13 (5):717–25.	2016	UK	Survey	Online (UK, USA, Germany, Italy)	Chronic/recalcitrant wounds
Metcalf D, Parsons D, Bowler IP. DEVELOPMENT OF A NEXT‐GENERATION ANTIMICROBIAL WOUND DRESSING. Acta medica Croatica: c̆asopis Hravatske Akademije Medicinskih Znanosti. 2016;70 (1):49–56.	2016	UK	Literature review, non‐ systematic (Clinical observation)	Not applicable	Chronic wounds
Metcalf DG, Bowler PG, Hurlow J. A clinical algorithm for wound biofilm identification. Journal of Wound Care. 2014;23 (3):137–42.	2014	UK	Literature review, non‐systematic (Algorithm proposal)	Not applicable	Chronic wounds
Metcalf DG, Parsons D, Bowler PG. Clinical safety and effectiveness evaluation of a new antimicrobial wound dressing designed to manage exudate, infection and biofilm. International Wound Journal. 2017;14 (1):203–13.	2017	UK	Case series	60 health care facilities (hospitals, clinics, nursing homes) and community settings	Venous leg ulcers, Mixed Arterial/Venous leg ulcers, Arterial ulcers, Pressure ulcers, Diabetic foot ulcers, Leg ulcers, Traumatic ulcers, Cyst, Other
Metcalf D, Parsons D, Bowler P. A next‐generation antimicrobial wound dressing: a real‐life clinical evaluation in the UK and Ireland. Journal of Wound Care. 2016;25 (3):132, 4–8.	2016	UK	Case series	8 healthcare facilities	Mixed arterial, venous leg ulcers, Venous leg ulcers, Diabetic foot ulcers, Pressure ulcers, Arterial ulcers, Leg ulcers, traumatic, cyst, other
Miller CC, Miller MK, Ghaffari A, Kunimoto B. Treatment of chronic nonhealing leg ulceration with gaseous nitric oxide: A case study. Journal of Cutaneous Medicine and Surgery. 2004;8 (4):233–8.	2004	Canada	Case report	Community/home	Venous leg ulcers
Min W. Antibacterial protease combined with silver dressing for the intervention of bacterial biofilm infection in chronic wounds. https://trialsearchwhoint/Trial2aspx?TrialID=ChiCTR1800019687. 2018.	2018	China	Randomised controlled trial protocol	Hospital	Chronic wounds
Morris C. Why biofilm‐based wound management is vital. Journal of Community Nursing. 2020;34 (5):8–9.	2020	UK	Editorial	Not applicable	Chronic wounds
Murphy C, Atkin L, Vega de Ceniga M, Weir D, Swanson T, Walker A, et al. Embedding Wound Hygiene into a proactive wound healing strategy. Journal of Wound Care. 2022;31 (Sup4a):S1‐S19.	2022	Canada	Consensus document	Not applicable	Hard to heal wounds
Oldfield R. Exuding venous leg ulcer with signs of biofilm. British Journal of Nursing (Mark Allen Publishing). 2017;26 (Sup20a):S24‐S5.	2017	UK	Case report	Not reported	Venous leg ulcers
Palomar Llatas F, Pastor Orduña MI, Bonías López J, Fornes Pujalte B, Sierra Talamantes C, Zamora Ortiz J, et al. Características y manejo del lecho de las heridas crónicas; Characteristics and management of the bed of chronic wounds. Ill Dermatol. 2018;12 (33):10–8.	2018	Spain	Literature review, non‐systematic	Not applicable	Chronic wounds
Pankhurst S. Heavily exuding venous leg ulcer with suspected biofilm. British Journal of Nursing (Mark Allen Publishing). 2017;26 (Sup20a):S29‐S30.	2017	UK	Case report	Leg ulcer clinic	Venous leg ulcers
Pedro I, Saraiva S. INTERVENÇÕES DE ENFERMAGEM NA GESTÃO DE BIOFILMES EM FERIDAS COMPLEXAS. Journal of Aging and Innovation. 2012;1 (6):78–88.	2012	Portugal	Systematic review	Not applicable	Chronic/complex wounds
Percival SL, Hill KE, Malic S, Thomas DW, Williams DW. Antimicrobial tolerance and the significance of persister cells in recalcitrant chronic wound biofilms. Wound Repair Regen. 2011;19 (1):1–9.	2011	UK	Literature review, non‐systematic	Not applicable	Chronic wounds
Percival SL, Hill KE, Williams DW, Hooper SJ, Thomas DW, Costerton JW. A review of the scientific evidence for biofilms in wounds. Wound Repair Regen. 2012;20 (5):647–57.	2012	UK	Literature review, non‐systematic	Not applicable	Chronic wounds, Diabetic foot ulcers
Percival SL, Vuotto C, Donelli G, Lipsky BA. Biofilms and wounds: An identification algorithm and potential treatment options. Adv Wound Care. 2015;4 (7):389–97.	2015	UK	Literature review, non‐systematic (algorithm proposal)	Not applicable	Chronic wounds
Phillips PL, Wolcott RD, Fletcher J, Schultz GS. Biofilms made easy. International Wound Journal. 2010;1 (3): 1–6.	2010	USA	Literature review, non‐systematic	Not applicable	Chronic wounds
Pinto GPNM. Biofilmes e feridas crónicas. Porto. Universidade Fernanto Pessoa; 2016.	2016	Portugal	Systematic review (master's thesis, algorithm proposal)	Not applicable	Chronic wounds
Carlos Poblete J, Juliany Lino Gomes S, Flávia Cristina Z, Thais R, Maria Helena Melo L. Biofilme e feridas crônicas: reflexões para o cuidado de enfermagem. Current Nursing Journal. 2017;81.	2017	Brazil	Integrative review	Not applicable	Chronic wounds
Price J, Boulton Z. Case 13: chronic painful ulcer on the heel of a diabetic foot. Journal of Wound Care. 2016;25 (3):S21.	2016	UK	Case report	Not applicable	Pressure ulcer on diabetic foot
Pupp G, Koivunen R. How To Assess The Bacterial Burden Of DFUs. Podiatry Today. 2012;25 (7):62–8.	2012	USA	Literature review, non‐systematic	Not applicable	Diabetic foot ulcers
Rama D, Fonseca B, Blanck M. 1a RECOMENDAÇÃO BRASILEIRA PARA O GERENCIAMENTO DE BIOFILME EM FERIDAS CRÔNICAS E COMPLEXAS. Sociedate Brasileira de Enfermagem em Feridas e Estética – SOBENFEE. São Gonçalo. 2018. http://hdl.handle.net/10400.26/27331	2018	Brazil	Practice recommendation	Not applicable	Complex/chronic wounds
Rodgers A, Watret L. How might biofilms affect dressing choice for the diabetic foot? Diabetic Foot Journal. 2008;11 (2):86.	2008	UK	Editorial	Not applicable	Diabetic foot ulcers
Roes C, Calladine L, Morris C. Biofilm management using monofilament fibre debridement technology: outcomes and clinician and patient satisfaction. Journal of Wound Care. 2019;28 (9):608–22.	2019	Germany	Case series	Real world routine care settings across all regions of the UK + web‐based survey	Leg ulcers, Pressure ulcers, Dehisced surgical ulcer, other
Salazar Trujillo MA. Management of complex wounds treatment with a hydrofiber dressing in a public hospital in Bogota (Colombia); Лечение сложных ран гидроволокнистыми перевязочными средствами в условиях городской больницы Боготы (Колумбия). Wounds and Wound Infections. 2021;8 (3):26–34.	2021	Colombia	Case series	Dept. of plastic surgery	Vascular ulcers, post‐traumatic ulcers, Diabetic foot ulcers, Pressure ulcers, post‐surgical oncological ulcers, Arterial ulcers, post‐surgical ulcers
Salazar Trujillo MA, Ortiz Rodriguez JE, Ospina AZ. Efectividad de un apósito de hidrofibra reforzada, con plata iónica al 1,2%, potenciado con EDTA y cloruro de bencetonio: casos de estudio. Journal of Wound Care. 2020;29 (Lat AM Sup1):1–12.	2020	Colombia	Case series	Wound clinic	Venous leg ulcers, Pressure ulcers, Arterial ulcers, Surgical site infections, Friction wounds, burns, facial (retinoblastoma), mastitis, traumatic, Seroma, fasciotomy
Sanjuan Sanchez MJ. REVISIÓN SISTEMÁTICA. ESTRATEGIAS TERAPÉUTICAS PARA LA GESTIÓN DEL BIOFILM BACTERIANO EN HERIDAS CRÓNICAS. Alicante: Universidad de Alicante; 2018.	2018	Spain	Systematic review (thesis)	Not applicable	Chronic wounds
Santos V, Santos AS, Menoita E. A ABORDAGEM DE BIOFILMES EM FERIDAS: ESTUDOS DE CASO. Journal of Aging and Innovation. 2013;2 (1):76–96.	2013	Portugal	Case series	NR	Venous leg ulcers, Diabetic foot ulcers, Pressure ulcers
Schultz G, Bjarnsholt T, James GA, Leaper DJ, McBain AJ, Malone M, et al. Consensus guidelines for the identification and treatment of biofilms in chronic nonhealing wounds. Wound Repair and Regeneration: official publication of the Wound Healing Society [and] the European Tissue Repair Society. 2017;25 (5):744–57.	2017	USA	Consensus guideline	Not applicable	Chronic wounds
Siaw‐Sakyi V. Identification and management of biofilm in chronic wounds. Journal of Community Nursing. 2018;32 (5):32–40.	2018	UK	Literature review, non‐systematic	Not applicable	Chronic wounds
St‐Cyr D. [Biofilms. What are they? Where are they? What impact do they have on wound care?]. Perspective Infirmière: Revue officielle de l'Ordre des Infirmières et infirmiers du Québec. 2011;8 (3):36–8.	2011	Canada	Literature review, non‐systematic	Not applicable	Chronic wounds
Swanson T. Innovations in the assessment and diagnosis of wound infection. Wounds International. 2011;2 (1):5–6.	2011	Australia	Literature review, non‐sysematic	Not applicable	Chronic wounds
Swanson T, Ousey K, Haesler E, Bjarnsholt T, Carville K, Idensohn P, et al. Wound Infection in Clinical Practice:Principles of Best Practice: International Consensus Update: Wounds International; 2022.	2022	Australia	Consensus document	Not applicable	Chronic wounds
Swanson T, Keast D, Bain K, Bain M. Preventing and treating infection in wounds: translating evidence and recommendations into practice. Wounds International. 2020;11 (4):82–7.	2020	Australia	Survey	Virtual, face to face, 19 countries	Chronic wounds
Swanson T, Haesler E, Angel D, Sussman G. IWII Wound infection in clinical practice consensus document 2016 update. Wound Practice & Research. 2016;24 (4):194–8.	2016	Australia	Editorial (Report on a consensus document update)	Not applicable	Wound infection (various aetiologies)
Swanson T. Understanding biofilm in practice: a global survey of health professionals. Journal of Wound Care. 2017;26 (8):426–40.	2017	Australia	Survey	Global (UK, other, Europe, Australia, New Zealand, USA, Canada, Japan, Middle East, Asia, South America)	Wounds
Temprano‐Andrés SA, Martínez‐Antón S. Aproximación a la importancia del biofilm en las heridas crónicas. Revisión bibliográfica. Enfermería Dermatológica. 2020;14 (39):23–8.	2020	Spain	Systematic review	Not applicable	Chronic wounds
Torregosa Jerez L. MÉTODOS DE DIAGNÓSTICO PARA LA IDENTIFICACIÓN DE BIOFILM EN HERIDAS CRÓNICAS. REVISIÓN SISTEMÁTICA. Alicante: Universidad de Alicante; 2015.	2015	Spain	Systematic review (thesis)	Not applicable	Chronic wounds
Anonymous. Biofilm as the Cause of Non‐Healing Wounds. Medsurg Nursing: Official journal of the Academy of Medical‐Surgical Nurses. 2015;24 (2):Suppl‐3.	2015	USA	Editorial	Not applicable	Non‐healing wounds
Vallejo A, Wallis M, McMillan D, Horton E. Use of low‐frequency contact ultrasonic debridement with and without polyhexamethylene biguanide in hard‐to‐heal leg ulcers: an RCT protocol. Journal of Wound Care. 2021;30 (5):372–9. Vallejo A, Wallis M, McMillan D. Use of low‐frequency contact ultrasonic debridement with and without polyhexamethylene biguanide in hard‐to‐heal leg ulcers: an RCT. Journal of Wound Care. 2022;31 (8):670–81.	2021/2022	Australia	Randomised controlled trial + Protocol	Community specialised wound clinic	Venous leg ulcers, arterial ulcers, Mixed arterial/venous leg ulcers
Vallejo A, Wallis M, Horton E, McMillan DJ. Low‐frequency ultrasonic debridement and topical antimicrobial solution Polyhexamethylene biguanide for use in chronic wounds: a case series. Wound Practice & Research. 2018;26 (1):4–13.	2018	Australia	Case series	Not reported	Venous leg ulcers
Vyas KS, Wong LK. Detection of Biofilm in Wounds as an Early Indicator for Risk for Tissue Infection and Wound Chronicity. Annals of Plastic Surgery. 2016;76 (1):127–31.	2016	USA	Literature review, non‐systematic	Not applicable	Chronic wounds
Walker M, Metcalf D, Parsons D, Bowler P. A real‐life clinical evaluation of a next‐generation antimicrobial dressing on acute and chronic wounds. Journal of Wound Care. 2015;24 (1):11–22.	2015	UK	Case series	33 Healthcare facilities	Venous leg ulcers, Traumatic ulcers, Pressure ulcers, Abscess, Cellulitis, Inflammation, Radiation, Rectal, Tumour
Webb R. A chronic case of confusion. Journal of Wound Care. 2017;26 (8):421.	2017	UK	Editorial	Not applicable	Chronic wounds
Wei D, Zhu XM, Chen YY, Li XY, Chen YP, Liu HY, et al. Chronic wound biofilms: diagnosis and therapeutic strategies. Chinese Medical Journal. 2019;132 (22):2737–44.	2019	China	Systematic review	Not applicable	Chronic wounds
Westgate S, Cutting K. The role of microbial biofilms in chronic and acute wounds. Nursing & Residential Care. 2011;13 (11):518–21.	2011	UK	Literature review, non‐systematic	Not applicable	Chronic and acute wounds
White RJ, Cutting KF. Wound biofilms‐are they visible? Journal of Wound Care. 2012;21 (3):140–1.	2012	UK	Letter	Not applicable	Chronic wounds
White R, Cooper R, Edwards‐Jones V. What is the role of biofilms in wound healing? Wounds UK. 2012;8 (2):20–4.	2012	UK	Editorial (Debate report)	Not applicable	Wounds
Widgerow AD. Persistence of the chronic wound—implicating biofilm. Wound Healing Southern Africa. 2008;1 (2):05–7.	2008	S. Africa	Literature review, non‐systematic	Not applicable	Chronic wounds
Wilson P, Gillen C, Hughes M. A clinical case series on the effectiveness of an enhanced ionic silver hydrofiber dressing in the management of diabetic foot ulceration. Diabetic Foot Journal. 2018;21 (4):239–46.	2018	Ireland	Case series	Podiatry‐led foot clinic in a large urban tertiary referral centre	Diabetic foot ulcers
Wolcott RD, Rhoads DD, Bennett ME, Wolcott BM, Gogokhia L, Costerton JW, et al. Chronic wounds and the medical biofilm paradigm. Journal of Wound Care. 2010;19 (2):45–6, 8–4650, 52–53.	2010	USA	Literature review, non‐systematic + Case series	Not reported	Venous leg ulcers, Traumatic (PAD)
Bjarnsholt T, Cooper R, Fletcher J, K. K‐M, Malone M, Schultz G, et al. World Union of Wound Healing Societies (WUWHS), Florence Congress, Position Document. Management of biofilm. Wounds International [Internet]. 2016.	2016	Denmark	Position document	Not applicable	Hard‐to‐heal wounds
Young L. Identifying infection in chronic wounds. Wound Practice & Research. 2012;20 (1):38–44.	2012	Australia	Literature review, non‐systematic	Not applicable	Chronic wounds

## Appendix B

Table of excluded full text reports.

**Table:**

	Reference	Reason for exclusion
1	Ammons MCB. Fuchs AL. Tripet BP. et al. Chronic diabetic wounds: Longitudinal profiling of the evolving microbiome and metabolic landscape in diabetic patients. FASEB Journal. 2017; https://doi.org/10.1096/fasebj.31.1_supplement.944.9	Irretrievable
2	Bello YM. Falabella AF. & Cazzaniga AL. Are biofilm present in human chronic wounds? Presented at the symposium on advanced wound care and medical research forum on wound repair in Las Vegas, NV, April 30–May 3 2001	Irretrievable
3	Brambilla R. Simone T. Giuseppina G. et al. Local control of bioburden and infection in mdrb. J Wound Care. 2020; 29: 276	Irretrievable
4	Cheng H. & Huang G. Research progress on bacterial biofilms in chronic wounds. Chinese Nursing Research. 2020; 34 (10): 1754–1758	Irretrievable
5	Chrysostomou D. Biofilm‐comparison of two methods of wound swabbing. J Wound Care. 2017; 26: 185	Irretrievable
6	Cogo A. Quint BJ. & Bignozzi CA. The clinical efficacy of a novel desiccant agent, debrichem in the treatment of chronic skin wounds: A case series of 50 patients. J WOUND CARE. 2020; 29: 63–64	Irretrievable
7	Colella R. New biotechnologies: the importance of biofilm management and wound bed preparation to promote tissue repair in the management of the diabetic foot. Case report. Italian Journal of Medicine. 2021; 15 (3): 26	Irretrievable
8	Liu J & Liu Y. Research advances on the formation mechanism and diagnosis of bacteria biofilms in chronic wounds. Chinese Journal of burns. 2021; 37 (7): 692–696	Irretrievable
9	Nakagami G. Evaluation of wound management based on biofilm detection at bedside: A randomised controlled trial. JPRN‐UMIN000029684. 2017 (Accessed 5 September 2022)	Irretrievable
10	Rastelli C. Nedjua B. Isaac CGJ. Et al. The use of Co2 Laser for the debridement of hard to heal wounds. J WOUND CARE. 2020; 29 (Suppl 7B): 173	Irretrievable
11	Rhoads D. Wolcott R. Cutting KF et al. (2007) Biofilms and management in chronic infected wounds. In McBain A. Allison D. Pratten J. et al. eds. Biofilms: coming of age. Cardiff, Bioline.	Irretrievable
12	Suleman L. & Chapp L. Biofilm‐infected pressure ulcers: Current knowledge and emerging treatment strategies. Reference details unknown.	Irretrievable
13	Swanson T. Wound infection and management: Playing detective with each dressing change. J WOUND CARE. 2020; 29: 118	Irretrievable
14	Thomas JG. Corum L & Mantlagh H. Chronic wounds: infectious diseases that won't go away. Wounds, gauzes and biofilms combine for an ugly triad. Advance for Administrators of the Laboratory. 2012; 21 (5): 18–20	Irretrievable
15	Unknown author. ¿Tenemos las herramientas necesarias para luchar contra el biofilm? (Do we have the necessary tools to fight biofilms)? Journal unknown; 32 (1): 14–5	Irretrievable
16	Unknown author. Biofilm and slough. Wounds UK. 2010; 6 (1): 162–163	Irretrievable
17	Unknown author. Biofilms and the role of debridement in chronic wounds. Wounds UK. 2010; 6 (1): 160–162	Irretrievable
18	Wolcott R. Dowd S. Kennedy J. et al. Biofilm based wound care. Advances in Wound Care. 2010; 1 (3): 311–318	Irretrievable
19	Wound Care Center. Biofilm based wound management. Available at www.woundcarecenter.net/CHAPTERTEXT.pdf (2006)	Irretrievable
20	Yu JA. & Gao XX. Bacterial biofilm and chronic wound infection. Chinese Journal of Burns. 2019; 35 (12): 842–847	Irretrievable
21	Amaral V. Silva NDS. Osis SL. Treatment of wound after amputation toes of the left foot in patients with diabetes mellitus. J WOUND CARE. 2017; 26 (suppl 6): 312	Wrong population
22	Serralta VW. Harrison‐Balestra C. Cazzinaga AL. et al. Lifestyles of bacteria in wounds: Presence of biofilm? Wounds. 2001; 13: 29–34	Wrong population
23	Heridas Crónicas: Biofilm y la importancia del Desbridamiento (Biofilm and the importance of debridement). Sociedad Iberoamericana de Información Cíentifica (SIIC) Buenos Aires. Biofilm y la Importancia del Desbridamiento	No concept data
24	Alkhatieb MT. Biofilm in the diabetic foot: an added challenge. Gerokomos. 2019; 30 (3): 157–160	No concept data
25	Amin O. Wright BJ. Schultz G. et al. Microbial biofilms and chronic wounds. Microorganisms. 2017; 5 (9):	No concept data
26	Ammons MCB. Anti‐biofilm strategies and the need for innovation in wound care. Recent Patents on Anti‐infective Drug Discovery. 2010; 5 (1): 10–17	No concept data
27	Armstrong DG. Edmonds ME. & Serena TE. Point‐of‐care fluorescence imaging reveals extent of bacterial load in diabetic foot ulcers. International Wound Journal. 2023; 20 (2): 554–566	No concept data
28	Ascenzioni F. Cloeckaert A. Di Domenico EG. et al. Editorial: Microbial biofilms in chronic and recurrent infections. Frontiers in Microbiology. 2021; 12: 1–3	No concept data
29	Attinger C. & Wolcott R. Clinically addressing biofilm in chronic wounds. Advances in Wound Care. 2012; 1 (3): 127–132	No concept data
30	Ayush R. Ankit R. Singh S. et al. Cadexomer versus povidone iodine dressing in chronic leg ulcers‐ a prospective study. Student's Journal of Health Research. 2023; 4 (3):	No concept data
31	Banu A. Noorul Hassan MM. Rajkumar J. et al. Spectrum of bacterial associated with diabetic foot ulcer and biofilm formation: A prospective study. Australasian Medical Journal. 2015; 8 (9): 280–285	No concept data
32	Barbara DA. Fabrizio O. Morais D'Autilio Margarida FL. et al. Infection's control and regenerative boos with silver sulfadiazine. J WOUND CARE. 2020; 29: 303	No concept data
33	Barker JC. Khansa I. & Gorddillo GM. A formidable foe is sabotaging your results: what you should know about biofilms and wound healing. Plast Reconstr Surg. 2017; 139 (5): 1184e—1194e	No concept data
34	Bartoszewicz M. Junka A. Dydak K. et al. OCENA SKUTECZNOŚCI OPATRUNKU URGOCLEAN AG WZGLĘDEM FORM BIOFILMOWYCH PATOGENÓW RAN PRZEWLEKŁYCH (Evaluation of the efficacy of UrgocleanAG dressing against biofilm forms of chronic wound pathogens). Leczenie Ran. 2018; 15 (2): 45–50	No concept data
35	Benavent E. Soldevila L. & Murilo O. Protocolo diagnóstico de las infecciones de úlceras del pie diabético (Diagnostic protocol for diabetic foot ulcer infections). Medicine. 2018; 12 (51): 3048–51	No concept data
36	Bertesteanu S. Triaridis S. Stankovic M. et al. Polymicrobial wound infections: Pathophysiology and current therapeutic approaches. International Journal of Pharmaceutics. 2014; 463: 119–126	No concept data
37	Bianchi T. Wolcott RD. Peghetti A. et al. Recommendations for the management of biofilm: A consensus document. 2016; 0 (0): 1–11	No concept data
38	Bianchi T. Peghetti A. & Pomponio G. Biofilm: It is time for consensus. J WOUND CARE. 2015; 24 (7): 291	No concept data
39	Bjarnsholt T. The role of bacterial biofilms in chronic infections. Acta Pathologica Microbiologica et Immunologica Scandinavica. 2013; 0 (136): 1–51	No concept data
40	Black CE. & Costerton JW. Current concepts regarding the effect of wound microbial ecology and biofilms on wound healing. Surg Clin N Am. 2010; 90: 1147–1160	No concept data
41	Boutli‐Kaspidou F. Delli F. Avogoustinaki N. et al. What are biofilms? Evaluation and management in open skin wounds. JEADV. 2006; 20: 735–767	No concept data
42	Brantley J. Park H. Sanchez PJ. et al. The use of a novel antimicrobial and purified native collagen matrix combination to manage bioburden and support healing in challenging wounds: A clinical evaluation. Wounds International. 2016; 7 (3): 40–45	No concept data
43	Brinkert D. Ali M. Naud M. et al. Negative pressure wound therapy with saline instillation: 131 patient case series. International Wound Journal. 2013; 10: 56–60	No concept data
44	Brognara L. Salmaso L. Mazzotti A. et al. Effects of probiotics in the management of infected chronic wounds: From cell culture to human studies. Curr Clin Pharmacol. 2020; 15 (3): 193–206	No concept data
45	Brown A. & Yorke M. Drawtex: Breaking the vicious circle of cellular and molecular imbalances. Br J Community Nurs. 2013: S42‐S49	No concept data
46	Caldas Arias L. Bacterias‐biofilms y resistencia antimicrobiana (Bacterial biofilms and antimicrobial resistance). Revista Facultad Ciencias de la Salud. Universidad del Cauca. 2015; 17 (1): 20–27.	No concept data
47	Carpenter S. Davis S. Fitzgerald R. et al. Expert recommendations for optimising outcomes in the management of biofilm to promote healing of chronic wounds. Wounds. 2016; 28 (Suppl 6): S1‐S20	No concept data
48	Carter MJ. & Myntti MF. Cost‐utility of a biofilm‐disrupting gel versus standard of care in chronic wounds: A Markov microsimulation model based on a randomised controlled trial. J WOUND CARE. 2022; 28 (7 N American suppl): S24‐S39	No concept data
49	Carville K. Howse L. Edmonson M. et al. Nurse practitioners and their use of low‐frequency ultrasound debridement in the management of chronic wounds. Wounds Practice and Research. 2018; 26 (3): 122–126	No concept data
50	Chang YJR. Perry J. & Cross K. Low‐Frequency ultrasound debridement in chronic wound healing: A systematic review of current evidence. Plastic Surgery. 2017; 25 (1): 21–26	No concept data
51	Chen V. Burgess JL. Verpile R. et al. Novel diagnostic technologies and therapeutic approaches targeting chronic wound biofilms and microbiota. Curr Dermatol Rep. 20 232; 11 (2): 60–72	No concept data
52	Cogo A. Quint BJ & Bignozzi CA. Restarting the healing process of chronic wounds using a novel dessicant: A prospective case series. Wounds. 2021; 33 (1): 1–8.	No concept data
53	Cole W. Human acellular dermal matrix paired with solver‐zinc coupled electroceutical dressing results in rapid healing of complicated diabetic wounds of mixed aetiology: a novel case series. Wounds: A Compendium of Clinical Research & Practice. 2016; 28 (7): 241–7	No concept data
54	Cooper R. Biofilms and wounds: Much ado about nothing? Wounds UK. 2010; 6 (4): 84–90	No concept data
55	Cooper R. Bjarnsholt T. & Alhede M. Biofilms in wounds: a review of present knowledge. J WOUND CARE. 2022; 23 (11): 570–582	No concept data
56	Cooper R. Chapter 6: Understanding biofilms. In Essential Wound Microbiology, Edwards‐Jones V. (ed). Oxford University Press, Oxford, 2016	No concept data
57	Crew JR. Varilla R. Rocas A. et al. Neutrophase with Sorbact dramatically enhances the speed of wound healing. Wound Repair & Regeneration. 2011; 19 (2): A19	No concept data
58	Cutting KC. & Percival SL. Biofilm management. Nurs Standard. 2009; 36: 389–95	No concept data
59	Cutting KF. Why use topical antiseptics? J WOUND CARE. 2011; 20 (Suppl 2):	No concept data
60	Da Conceição Mota M. Cabral de Melo S. & Pereira Costa T. Estrategia de gestaode biofilmes em feridas cronicas: uma revisao dea literatura (Biofilm management strategy in chronic wounds: a literature review). J. Tissue Regeneration & Healing. 2012: 10–18	No concept data
61	DesJardins H. Char S. Marasco P. et al. Efficacy of hydromechanical therapy in nonhealing, chronic wounds as a cost and clinically effective wound care modality. Wounds: A Compendium of Clinical Research & Practice. 2021; 33 (11): 296–303	No concept data
62	Ding X. Tang Q. Xu Z. et al. Challenges and innovations in treating chronic and acute wound infections: from basic science to clinical practice. Burns & Trauma. 2022; 10: tkac014	No concept data
63	Dinh T. & Lunborg M. Assessing the impact of biofilm and the microbiome in diagnosing and treating DFUs. Podiatry today. 2016; 29 (8): 16–20	No concept data
64	Esquiva Castillo L. Validez y fiabilidad de los instrumentos para la valoración clínica de infección en heridas crónicas: una revisión sistemática (Validity and reliability of instruments for the clinical assessment of infection in chronic wounds: a systematic review). Alicante. Facultad de Ciencias de la Salud, Universidad de Alicante; 2023.	No concept data
65	Evelhoch SR. Biofilm and chronic nonhealing wound infections. The Surgical Clinics of North America. 2020; 100 (4): 727–732	No concept data
66	Fazli M. Bjarnsholt T. Kirketerp‐Moller K. et al. Non‐Random distribution of *Pseudomonas aeruginosa* and *Staphylococcus aureus* in chronic wounds. J Clin Microbiol. 2009;47 (12): 4084–4089	No concept data
67	Formosa C. & Vella L. Characteristics predicting the outcome in individuals with diabetic foot ulcerations. Diabetic Medicine. 2016; 33: 52	No concept data
68	Gajula B. Munnamgi S. & Basu S. How bacterial biofilms affect chronic wound healing: a narrative review. International Journal of Surgery: Global Health. 2020; 3 (2): e16	No concept data
69	Goss SG. Alcantara SD. Gendics C. et al. Sharp debridement reduces biofilm more robustly than planktonic bacteria in chronic venous leg ulcers. Wound Repair & Regeneration. 2014; 22 (2): A42	No concept data
70	Greener M. UrgoClean Ag: evidence base and mode of action. British Journal of Nursing. 2017; 26: S12‐S15	No concept data
71	Håkans E. Sårvård med fokus på sekret och biofilm: en litteraturstudie (Wound care with a focus on secretions and biofilm: a literature study). Yrkeshögskolan Novia, 2018	No concept data
72	Hall MR. McGillicuddy E. & Kaplan LJ. Biofilm: basic principles, pathophysiology and implications for clinicians. Surgical Infections. 2014; 15 (1): 1–7	No concept data
73	Hampton S. Caring for sloughy wounds. J Community Nurs. 2005; 19 (4): 30–34	No concept data
74	Hermans MH. A retrospective study: rapid removal of biofilm and necrosis with a hygroscopic chemical debriding compound. Journal of Burn Care & Research. 2022; 43: S207.	No concept data
75	Hernández‐ Martínez‐Esparza E. Santesmases‐Masana R. Román E. et al. Prevalence and characteristics of older people with pressure ulcers and leg ulcers in nursing homes in Barcelona. Journal of Tissue Viability. 2021; 30: 108–115	No concept data
76	Høiby N. Bjarnsholt T. Moser C. et al. ESCMID guideline for the diagnosis and treatment of biofilm infections 2014. Clinical Microbiology and Infection. 2015; 21: S1‐S25	No concept data
77	Honorato‐Sampaio K. Martins Guedes CA. de Araújo Nogueira Lima VL. et al. Bacterial biofilm in chronic venous ulcer. The Brazilian Journal of Infectious Diseases. 2014; 18 (3): 350–351	No concept data
78	Hurlow J. Couch K. Laforet K. et al. Clinical biofilms: A challenging frontier in wound care. Advances in Wound Care. 2015; 4 (5): 295–301	No concept data
79	Hurlow J. Response to White and Cutting critique. J WOUND CARE. 2012; 21 (4): 198	No concept data
80	Husain FM et al. (2021). MRSA, EBSL, and biofilm formation in diabetic foot ulcer infections. In Zubair M. Ahmat J. Malik A. Talluri MR. (eds) Diabetic foot ulcer. Springer, Singapore.	No concept data
81	Jones EM. Cochrane CA. & Percival SL. The effect of pH on the extracellular matrix and biofilms. Advances in Wound Care. 2015; 4 (7): 431–439	No concept data
82	Kalkanci A. & & Güzel Tunçcan Ö. Biyofilm İlişkili Enfeksiyonlar: Tanı, Tedavi ve Korunma (Biofilm‐related infection: diagnosis and treatment). Mediterr J Infect Microb Antimicrob. 2019; 8: 18	No concept data
83	Karaeng R. Gambaran Perawatan luka pada Tn. AA dengan Ulkus Vena (Venous Ulcer) di klinik perawatan luka ETN Centre Makassar: Laporan Kasus (Overview of wound care in Mr. AA with Venous Ulcer at ETN Center Makassar wound careclinic: Case Report) 2021; Laporan Kasus. Skripsi thesis, universitas Hasanuddin. http://repository.unhas.ac.id:443/id/eprint/2929 (Accessed 5 September 2022)	No concept data
84	Kim D. William NI. Moore J. et al. Clinical assessment of a biofilm‐disrupting agent for the management of chronic wounds compared with standard of care: a therapeutic approach. Wounds: A compendium of clinical research and practice. 2018; 30 (5): 120–130	No concept data
85	Kingsley *A. Lewis* A. & White R. Debridement and wound biofilms. J WOUND CARE. 2011; 20 (6): 286	No concept data
86	Kirketerp‐Moller K. Zulkowski K. & James G. Chronic wound colonisation, infection and biofilms. In Bjarnsholt et al. Eds, Biofilm Infections. Springer, 2001	No concept data
87	Kirketerp‐Møller K. & Gottrup F. Bakteriel biofilm I kroniske sår (Bacterial biofilms in chronic wounds). Ugeskr Læger. 2009;17 (13): 1097	No concept data
88	Lantis J. Schultz G. Edmondson‐Jones M. et al. Effects of cadexomer iodine on biofilm in diabetic foot ulcers: a pilot study. J WOUND CARE. 2017; 26: 360	No concept data
89	Lavery LA. Bhavan K. & Wukich DK. Biofilm and diabetic foot ulcer healing: all hat and no cattle. Annals of Translational Medicine. 2019; 7 (7): 159	No concept data
90	Le L. Baer M. Briggs P. et al. Diagnostic accuracy of point‐of‐care fluorescence imaging for the detection of bacterial burden in wounds: results from the 350‐patient fluorescence imaging assessment and guidance trial. Advances in Wound Care. 2021; 10 (3): 123–136	No concept data
91	Ligonenko OV. Digtyar II. Chorna IO. et al. ВИКОРИСТАННЯ СИСТЕМНИХ ЕНЗИМОПРЕПАРАТІВ В ЯКОСТІ БУСТЕР‐ТЕРАПІЇ ДЛЯ БОРОТЬБИЗ МІКРОБНИМИ БІОПЛІВКАМИ ХРОНІЧНИХ РАН (A victory range of systemic enzyme preparations as a booster therapy for fighting with microbial bioples of chronic wounds). Актуальні проблеми сучасної медицини. 2013; 13 (1[41]): 216–217	No concept data
92	Loudon H. Part 2: Bacterial biofilm and the infection prevention “balancing act”: the case for using a polyhexamethylene biguanide solution in chronic and complex wound management. Professional Nursing Today. 2016; 20 (2): 42–45	No concept data
93	Manci KA. Kirsner RS. & Ajdic D. Wound biofilms: lessons learned from oral biofilms. Wound Repair and Regeneration. 2013; 21 (3): 352–62	No concept data
94	Marmolejo VL. Biofilm and DFUs: What the literature reveals. Podiatry Today. 2021; 34 (8): 1	No concept data
95	Martinez‐Pizarro S. Iodinated cadexomer iodine in the treatment of bacterial biofilms in chronic ulcers. Public Health of Mexico. 2020; 62 (4):	No concept data
96	Mazzanti S. & Rivaldi R. Use of silver sulfadiazine 1% cream between colonisation and infection in the skin lesions. J WOUND CARE. 2017; 26: 362	No concept data
97	McGuire J. & Zhou Z. Improving outcomes with better wound bed prep: Here's how to lower infection and amputation rates. Podiatry Management. 2022; 41 (6): 75–79.	No concept data
98	McReynolds S. Biofilms and practical wound care. World Council of Enterostomal Therapists Journal. 2005; 25 (1): 26–30	No concept data
99	Menoita E. Santos V. Gomes C. et al. Role of biofilms in chronic wounds. Journal of Aging and Innovation. 2012; 1 (2): 33–42	No concept data
100	Mihai MM. Holban AM. Giurcaneanu C. et al. Microbial biofilms: impact on the pathogenesis of periodontitis, cystic fibrosis, chronic wounds and medical device‐related infections. Current Topics in Medicinal Chemistry. 2015; 15 (6): 1552–76	No concept data
101	Milne C. Step it up! Using an integrated stepwise approach to achieve outcomes in the patient with diabetic lower extremity wounds. WOCN Society's 49th annual conference, Salt Lake City, Utah, May 19–23, 2017. Journal of Wound Ostomy & Continence Nursing. 2017; 44: S51	No concept data
102	Milne CT. An evaluation of a novel antimicrobial topical spray to reduce bioburden. Scientific and clinical abstracts from WOCNext 2020 Reimagined, June 5–7, 2020. Journal of Wound, Ostomy, & Continence Nursing. 2020; 47: S31‐S32	No concept data
103	Milne J. A research roundup of recent papers relevant to wound care. Wounds UK. 2015; 11 (1): 88–89	No concept data
104	Milne J. A research roundup of recent papers relevant to wound care. Wounds UK. 2018; 14 (5): 136	No concept data
105	Moser C. Jensen PO. Thomsen K. et al. Immune responses to *Pseudomonas aeruginosa* biofilm infections. Frontiers in Immunology. 2021; 12: 625597	No concept data
106	Moser C. Pedersen HT. Lerche CJ. et al. Biofilms and host response—helpful or harmful. APMIS: Acta Pathologica, Microbiologica, et Immunologica Scandinavica. 2017; 125 (4): 320–338	No concept data
107	Percival SL & Rogers CA. (2005) The significance and role of biofilms in chronic wounds. In McBain A. Allison D. Pratten J. et al. eds. Biofilms: persistence and ubiquity. Cardiff, Bioline 2005	No concept data
108	Murphy C. Atkin L. Dissemond J. et al. Defying hard‐to‐heal wounds with an early antibiofilm intervention strategy: ‘Wound hygiene’. J WOUND CARE. 2019; 28 (12): 818–822	No concept data
109	Murphy C. Wound hygiene: Stage 1‐cleanse. J WOUND CARE. 2020; 29 (3): S11‐S13	No concept data
110	Nakagami G. Novel point‐of‐care biofilm detection system for realising biofilm‐based wound care. J WOUND CARE. 2020; 29: 66	No concept data
111	Nakagami G. Schultz G. Gibson D. et al. Biofilm detection by wound blotting can predict slough development in pressure ulcers: A retrospective observational study. J WOUND CARE. 2017; 26: 149	No concept data
112	Nascimento LD. Lopes ACP. Teixeira MM. et al. Clinical and microbiological profile of diabetic foot ulcers infected with *Staphylococcus aureus* in a regional general hospital in Bahia, Brazil. The International Journal of Lower extremity Wounds. 2021: 15347346211050771	No concept data
113	Neut D. Tijdens‐Creusen EJA. Bulstra SK. et al. Biofilms in chronic diabetic foot ulcers: a study of 2 cases. Acta Orthopaedica. 2011; 82 (3): 383–5.	No concept data
114	Parekh J. Biofilm study in diabetic foot ulcer. 2021. https://www.cochranelibrary.com/central/doi/10.1002/central/CN‐02350287/full (Accessed 08 Feb 2022)	No concept data
115	Percival SL. & Bowler PG. Biofilms and their potential role in wound healing. Wounds: A Compendium of Clinical Research and Practice. 2004; 16 (7): 234–240	No concept data
116	Percival SL. & Suleman L. Slough and biofilm: removal of barriers to wound healing by desloughing. J WOUND CARE. 2015; 24 (11): 498–10	No concept data
117	Percival SL. McCarty S. Hunt JA. et al. The effects of pH on wound healing, biofilms, and antimicrobial efficacy. Wound Repair and Regeneration. 2014; 22: 174–186	No concept data
118	Phillips P. Yang Q. Gibson D. et al. Assessing the bioburden in poorly healing wounds. International Wound Journal. 2011; 2 (2): 17–19	No concept data
119	Potera C. Biofilms are key challenge in treating chronic wounds. Microbe. 2010; 5 (10): 414	No concept data
120	Pugazhendhi SH. & Prasanth Dorairaj A. Appraisal of biofilm formation in diabetic foot infections by comparing phenotypic methods with the ultrastructural analysis. The Journal of Foot and Ankle Surgery. 2018; 57 (2): 309–315	No concept data
121	Rhoads DD. Wolcott RE. Cutting KF. et al. Evidence of biofilms in wounds and potential ramifications. In Gilbert P. Allison D. Brading M. et al. Eds. Biofilms: coming of age. Manchester: BioLome	No concept data
122	Rodrigues CF. Kaushik K. & Light C. et al. Biofilms in wounds: New advances in therapy and in healing management. Biomedicines. 2021; 9 (193):	No concept data
123	Rucigaj TP. Biofilm and our clinical experience. Acta Medica Croatica. 2016; 70 (1): 57–9	No concept data
124	Sanjaya, Sri Hepti Sutiba (2020) EFEKTIFITAS PERAWATAN LUKA DENGAN MENGGUNAKAN TEKNIK MODEREN DRESSING DALAM PENANGANAN DIABETIC FOOT ULCER DI KLINIK ISAM CAHAYA: LAPORAN KASUS (Effectiveness of wound treatment using modern dressing techniques in handling diabetic foot ulcers at Isam Cahaya clinic: case report.). Skripsi thesis, universitas Hasanuddin.	No concept data
125	Schultz G. & Wolcott RD. Wound infection and diagnostics in practice: what is emerging? Wounds International. 2013; 4 (1): 4–6	No concept data
126	Schultz G. Audio lecture I. Battling biofilms: Winning the war in wounds. Foot & Ankle Quarterly—The Seminar Journal. 2020; 31 (1): 63–66.	No concept data
127	Schwarzer S. James GA. Goeres D. et al. The efficacy of topical agents used in wounds for managing chronic biofilm infections: a systematic review. The Journal of Infection. 2020; 80 (3): 261–270	No concept data
128	Schwarzer S. The efficacy of topical agents managing chronic wound biofilm infections: a systematic review. Wounds Practice & Research. 2022; 30 (4): 244–245	No concept data
129	Seyer D. Garde VL. Martino PD. et al. Wound Repair and Regeneration. 2011; 19 (5): A91	No concept data
130	Shah J. Ace your certification exam: biofilm‐based‐wound care. The Journal of the American College of Wound Specialists. 2011; 3 (4): 97–8	No concept data
131	Singh VJ. & Barbul A. Bacterial biofilm in wounds. Wound Repair and Regeneration. 2008; 16 (1): 1	No concept data
132	Sinozic T. Katic M. & Kovacevic J. Personalised approach to patient with chronic wound in family medicine. Acta Medica Croatica. 2016; 70: 105–10	No concept data
133	Skrlin J. Impact of biofilm on healing and a method for identifying it in the wound. Acta Medica Croatica. 2016; 70: 29–32	No concept data
134	Špoljar S. Treatment of venous ulcers of the entire shin in the patient with severe chronic renal insufficiency. J WOUND CARE. 2017; 26 (Suppl 6): 288	No concept data
135	Steinberg J. & Siddiqui F. The chronic wound and the role of biofilm. Podiatry Management. 2011; 30 (6): 181–190	No concept data
136	Stevenson P. & Schultz G. 2019 International consensus includes biofilm treatment as new standard of care. Ostomy Wound Manage. 2019; 65 (7): 7–9	No concept data
137	Suryaprakash A. Tejaswini V. Girish K. et al. Efficacy of honey dressing versus mechanical debridement in healing of ulcers with biofilms. A comparative study. JKIMSU. 2018; 7 (2): 49–55	No concept data
138	Swanson T. Ousey K. Haesler E. et al. An updated wound infection continuum from the wound infection in clinical practice consensus document 2022. Wounds Australia Conference, 14–17 September 2022, Sydney, Australia. Wounds Practice & Research. 2022; 30 (4): 233.	No concept data
139	Szkiler E. ZAKAŻENIE RAN I OWRZODZEŃ—POSTĘPOWANIE W PRAKTYCE PIELĘGNIARSKIEJ (Infections of wounds and ulcers‐management in nursing practice). Nursing in Anaesthesiology & Intensive care. 2019; 5 (2): 47–52	No concept data
140	Taha Alkhatieb M. Biofilm en el pie diabético: un reto añadido (Biofilm in the diabetic foot: An added challenge). Gerokomos. 201 930 (3): 157–160	No concept data
141	Thomson CH. Biofilms: do they affect wound healing? International Wound Journal. 2011; 8 (1): 63–7	No concept data
142	Unknown author. A passion for wound care. Australian Nursing Journal. 2013; 20 (7): 31	No concept data
143	Unknown author. An interview with Jaqui Fletcher. British Journal of Community Nursing. 2010; 15:S30	No concept data
144	Unknown author. Silver hydrofibrate versus collagenase curative in the control of venous ulcer infection: A randomised clinical trial. International Clinical Trials Registry Platform. 2023; RBR‐4kkq2h (Accessed 22 September 2022)	No concept data
145	Unknown author. Treatment of chronic wounds. Clinicaltrials.gov. 2012; NCT01646502	No concept data
146	Unknown author. Visualising biofilms in foot wounds. Diabetic Foot Journal. 2015; 18 (1): 51	No concept data
147	Vallabha T. Ragate A. Sindgikar V. et al. Is honey an answer for eradication of biofilms? Indian Journal of Surgery. 2019; 81 (2): 144–149	No concept data
148	Vella L. The association between baseline characteristics and the outcome of foot ulceration in a Maltese population with diabetes. 2015, University of Malta; Faculty of Health Sciences, Department of Podiatry	No concept data
149	Wang J. Chen XY. Zhao Y. et al. pH‐Switchable antimicrobial nanofiber networks of hydrogel eradicate biofilm and rescue stalled healing in chronic wounds. ACS Nano. 2019; 13: 11686–11 697	No concept data
150	Webb R. & Cutting K. Biofilm: the challenge. J WOUND CARE. 2014; 23 (11): 519	No concept data
151	Webb R. Hard‐to‐heal wounds: TIMERS for action. J WOUND CARE. 2019; 28 (3): 131	No concept data
152	Webb R. Skin integrity and infection prevention Las Vegas: don't bust on biofilm, bet on dHACM. J WOUND CARE. 2018; 27 (11): 764–766	No concept data
153	White R. & Cooper R. Biofilms in wounds: the chicken or the egg. J WOUND CARE. 2014; 23 (8): 425	No concept data
154	White RJ. Cutting K. & Cooper R. The diagnosis of biofilms in wounds. J WOUND CARE. 2016; 25 (1): 56	No concept data
155	White W. Sharp wound debridement in the management of recalcitrant, locally infected chronic venous leg ulcers: a narrative review. Wounds Practice & Research. 2011; 19 (4): 222–228	No concept data
156	Wiegand C. Rutge L. Cutting K. et al. The efficacy of non‐thermal gas plasma in the treatment of diabetic foot ulcers stalled by subclinical, biofilm‐related wound infection. Wounds International. 2022; 13 (4): 14–21	No concept data
157	Wilkins M. Hall‐Stoodley L. Allan RN. et al. New approaches to the treatment of biofilm‐related infections. Journal of Infection. 2014; 69: S47‐S52	No concept data
158	Witowska I. & Mitura K. Is the presence of biofilm the main factor that keeps us away from successful wound healing—report of a case series treated with hydrofiber silver dressings. J WOUND CARE. 2017; 26 (Suppl 6): 347	No concept data
159	Wolcott R. & Cox S. The effects of a hydroconductive dressing on wound biofilm. Wounds: A Compendium of Clinical Research & Practice. 2012 (September): 14–16	No concept data
160	Wolcott R. Are chronic wounds chronic infections? J WOUND CARE. 2016; 25 (1): S3	No concept data
161	Wolcott R. Practical clinical management of wound biofilm. J WOUND CARE. 2017; 26: 40	No concept data
162	Wolcott RD. & Cox S. More effective cell‐based therapy through biofilm suppression. J WOUND CARE. 2013; 22 (1): 26–31	No concept data
163	Wolcott RD. & Dowd S. The role of biofilms: Are we hitting the right target? Plastic & Reconstructive Surgery. 2011; 127 (1S): 28S—35S	No concept data
164	Wolcott RD. & Rhoads DD. A study of biofilm‐based wound management in subjects with critical limb ischaemia. J WOUND CARE. 2008; 17 (4): 145–155	No concept data
165	Wolcott RD. Biofilms cause chronic infections. J WOUND CARE. 2017; 26 (8): 423–425	No concept data
166	Wolcott RD. Kennedy JP. & Dowd SE. Regular debridement is the main tool for maintaining a healthy wound bed in most chronic wounds. J WOUND CARE. 2009; 18 (2): 54–56	No concept data
167	Wolcott RD. Lopez‐Leban F. Divakar Kiran M. et al. (2011). Wound healing by an anti‐Staphylococcal biofilm approach. In Flemming HC. et al. (eds) Biofilm Highlights, Springer series on Biofilms 5. Springer‐Verlag, Berlin Heidelberg	No concept data
168	Wolcott RD. Rhoads DD. & Dowd SE. Biofilms and chronic wound inflammation. J WOUND CARE. 2008; 17 (8): 333–341	No concept data
169	Woo KY. Alam T. & Marin J. Topical antimicrobial toolkit for wound infection. Surgical Technology International. 2014; 25: 45–52	No concept data
170	Woo KY. Physician's knowledge and attitudes in the management of wound infection. International Wound Journal. 2016; 13 (5): 600–4	No concept data
171	Woods EJ. Davis P. Barnett J. et al. (2010). Wound healing, immunology and biofilms. In Percival S. & Cutting K. (eds) Microbiology of wounds. CRC Press	No concept data
172	Wound healing and management node group. Evidence Summary: Wound infection: Biofilms defined and described. Wounds Practice & Research. 2012; 20 (4): 187–189	No concept data
173	Wound healing and management node group. Evidence summary: Wound infection: iodophores and biofilms. Wound Practice & Research. 2013; 21 (2): 8687	No concept data
174	Wu YK. Cheng NC & Cheng CM. Biofilms in chronic wounds: Pathogenesis and diagnosis. Trends in Biotechnology. 2019; 37 (5): 505–517	No concept data
175	Yadav H. Biofilms against pathogens for wound healing. Zenodo. 2016. https://zenodo.org/record/55725#.Y1aIhnbMKUk. (Accessed 24 October 2022)	No concept data
176	Yang S. Hu L. Han R. et al. Neuropeptides, inflammation, biofilms and diabetic foot ulcers. Experimental and Clinical Endocrinology and Diabetes. 2022; 130: 439–446	No concept data
177	Yarets Y. & Rubanov L. The identification of superficial chronic wound infection: Applicable clinical and laboratory diagnostic tests. J WOUND CARE. 2017; 26 (Suppl 6): 482	No concept data
178	Yarets Y. Effective biofilm removal and changes in bacterial biofilm building capacity after wound debridement with low‐frequency ultrasound as part of wound bed preparation before skin grafting. Chronic Wound Care Management & Research. 2017; 4: 554–64	No concept data
179	Young KA. Jin HE. Suk PH. et al. The effect of manuka honey in chronic wounds: A case study. J WOUND CARE. 2017; 26 (Suppl 6): 216	No concept data
180	Zubair m. Malik A. & Ahmad J. Incidence, risk factors for amputation among patients with diabetic foot ulcer in a North Indian tertiary care hospital. The Foot. 2012; 22 (1): 24–30	No concept data
